# A New Remedy in Erysipelas

**Published:** 1866-10

**Authors:** H. B. Withers

**Affiliations:** Rantoul, Ill.


*A New Remedy in Erysipelas.* By H. B. Withers, M. D., of Rantoul, Ill.

In May, 1864, while observing the effects of iodide of potassium in a form of urticaria, occasioned by the administration of copaiba, I was led to suspect that it would prove a useful remedy in erysipelas. Acting upon this supposition, I immediately commenced giving it to a patient then under my care, in whose case all the vaunted specifics had most signally failed. She improved at once, and was speedily cured. Since that date, I have used it in about thirty cases with perfect success.

It arrests the disease in from twelve to thirty-six hours. I usually give ten grains every two hours, observing closely the effect. As soon as the disease begins to subside, I discontinue the medicine. I use no external application whatever, but simply keep the parts covered and moist. I do not recommend it as a specific, but wish merely to bring it to the notice of the profession as an exceedingly valuable medicine in this disease.

